# Turning around an ailing district hospital: a realist evaluation of strategic changes at Ho Municipal Hospital (Ghana)

**DOI:** 10.1186/1471-2458-10-787

**Published:** 2010-12-24

**Authors:** Bruno Marchal, McDamien Dedzo, Guy Kegels

**Affiliations:** 1 Department of Public Health, Institute of Tropical Medicine-Antwerp, Nationalestraat 155, B-2000 Antwerp (Belgium; 2 Human Resources Directorate, Ghana Health Service, Accra, Ghana

## Abstract

**Background:**

There is a growing consensus that linear approaches to improving the performance of health workers and health care organisations may only obtain short-term results. An alternative approach premised on the principle of human resource management described as a form of 'High commitment management', builds upon a bundles of balanced practices. This has been shown to contribute to better organisational performance. This paper illustrates an intervention and outcome of high commitment management (HiCom) at an urban hospital in Ghana. Few studies have shown how HiCom management might contribute to better performance of health services and in particular of hospitals in low and middle-income settings.

**Methods:**

A realist case study design was used to analyse how specific management practices might contribute to improving the performance of an urban district hospital in Ho, Volta Region, in Ghana. Mixed methods were used to collect data, including document review, in-depth interviews, group discussions, observations and a review of routine health information.

**Results:**

At Ho Municipal Hospital, the management team dealt with the crisis engulfing the ailing urban district hospital by building an alliance between hospital staff to generate a sense of ownership with a focus around participative problem analysis. The creation of an alliance led to improving staff morale and attitude, and contributed also to improvements in the infrastructure and equipment. This in turn had a positive impact on the revenue generating capacity of the hospital. The quick turn around in the state of this hospital showed that change was indeed possible, a factor that greatly motivated the staff.

In a second step, the management team initiated the development of a strategic plan for the hospital to maintain the dynamics of change. This was undertaken through participative methods and sustained earlier staff involvement, empowerment and feelings of reciprocity. We found that these factors acted as the core mechanisms underlying the changes taking place at Ho Municipal Hospital.

**Conclusions:**

This study shows how a hospital management team in Ghana succeeded in resuscitating an ailing hospital. Their high commitment management approach led to the active involvement and empowerment of staff. It also showed how a realist evaluation approach such as this, could be used in the research of the management of health care organisations to explain how management interventions may or may not work.

## Background

Over the last 20 years, a number of strategies aimed at improving the performance of health services have been implemented in low- and middle-income countries (LMIC). Each strategy has had its specific perspective and focused on one particular issue: quality improvement, performance management, building learning organisations, innovation diffusion, etc. [1]. While success has been reported in some cases, there is increasing acknowledgement that to improve performance of health workers and health care organisations, approaches that deal with one problem, mostly fall short or obtain only short-term results.

These singular approaches stand in sharp contrast with the concept of high commitment management, an approach that is well developed in the domain of Human Resource Management (HRM). High commitment management holds that implementing balanced bundles of HRM practices leads to higher staff attraction and retention, and often to higher organisational performance. Such approaches are described under different names: high involvement work practices [2,3], high performance work systems [4], high commitment management [5] and high performance management [6]. These labels cover approaches based on similar basic assumptions [4]. For reasons of simplicity, we will use the term high commitment management (HiCom) practices throughout this text.

The key elements of HiCom may be summarised as follows:

(1) Bundles of management practices are more effective than singular practices [7].

(2) Key practices in HiCom include: selective staffing, self-managed teams, decentralised decision-making, attention for training and job flexibility, open communication and performance-contingent remuneration [4]. Pfeffer and Veiga add participative decision-making and efforts at the reduction of status differentials to the list [5].

(3) Effective bundles are balanced. They display good internal coherence (internal fit): bundles of management practices are synergistic, i.e. they reinforce each other and do not cancel each other out. They display good external coherence (external fit): good fit between the practices and the type of staff, the organisational mission/task and the type of organisation [8,9]. Well-balanced management approaches combine tight rules and procedures (command-and-control features) with loose, autonomy facilitating practices (empowerment of professionals).

The mechanisms that underlie such practices, or in other words the ways in which such bundles work, have not been studied well in LMIC. However, the literature from industrialised countries has identified leadership style, the internal social structure, the organisational climate and culture as important mechanisms.

Since HiCom maintains that it is not only the number of practices, but also how they are implemented that counts, leadership style may play an important role in the effect of high commitment management. In this respect, the concept of engaging leadership is an interesting one. It makes an explicit link between leadership and staff commitment. Alimo-Metcalfe and colleagues for example define engagement as "a positive attitude held by the employee towards the organisation and its values". Distributed or nearby-leadership stimulates such engagement: "*It is a model that is characterized by a strong sense of inclusiveness, in which leadership is seen as being 'distributed' throughout all levels of the organization*" [10]. Engaged leadership has shown to have a positive impact on motivation, commitment, absenteeism, staff turn-over and organisational performance in the private sector. Bradley & Alimo-Metcalfe obtained similar results in a study of leadership in multi-disciplinary mental health teams in the UK [11]. In HiCom, the elements of open communication and participative decision-making are in line with this perspective on leadership.

A second mechanism is the internal social structure, or the nature of relations between managers and workers. This has an impact on staff attitudes and behaviour in terms of task behaviour, absenteeism, staff turn-over and organisational citizenship behaviour [4,12]. HiCom's characteristics of reducing status differentials and decentralising decision-making power has an influence on the internal social structure of the organisation.

A third mechanism includes organisational climate and organisational culture. These could be considered as pathways through which HiCom may have a long-lasting effect. The *organisational climate *is defined as "the atmosphere that employees perceive is created in their organisation, by practices, procedures and rewards" [13]. HiCom practices have been shown to lead to a positive organisational climate in which reciprocity is central [14,15]. If leaders can translate their vision into practices that inspire others, their values and principles may be taken up by other staff, which may lead to shared ways of thinking and commitment that supports a strong *organisational culture *[16,17].

Despite the body of knowledge developed in industrialised countries, few studies have been carried out in LMIC settings on how good management of a health workforce can contribute to better performance and accountability of health services [18]. Only a handful of studies describe some of the challenges faced by health service managers *or health workers,) *and their (management) interventions from their perspective [19-24]. Even fewer studies explicitly explore the link between organisational management and performance. Couper and colleagues for example, compare differences in management style and organisational culture to explain varied performances of two first line services [25], while Puoane et al. examine why 4 hospitals differ in their nutritional programme outcomes by analysing organisational support and management [26]. Our previous work shows how HiCom practices may be well suited to the management of health workers [27] and that such approaches have been applied with positive results in Ghana [28].

The study we report on here is part of a longitudinal series of case studies in Ghana and Tanzania examining the links between management and the performance of hospitals. The findings of our previous case studies are generally in line with the bundle approach to management of performance. Our research has found that balanced bundles of health workforce management practices are more likely to increase organisational performance than singular or linear interventions [28].

This paper presents the findings of a study that focused on an ailing urban district hospital in Ghana. We chose Ho Municipal Hospital (HMH) as a case, because it is an interesting positive deviant case: its management team succeeded in improving the performance over a relatively short period of time. The research questions were as follows:

(1) Which strategies were implemented to improve the performance?

(2) What were the results in terms of health service provision?

(3) How do staff members perceive these practices (the organisational climate) and what were the HRM outcomes in terms of staff commitment?

(4) What made this intervention work?

The paper is structured as follows. We present in the Methods section the realist evaluation methodology and the middle range theory that formed the initial hypothesis of this study. We then present the results and the analysis. In the discussion, we frame our findings in the larger picture of the management literature and compare them with the findings of our previous case studies. We end with a revision of our initial middle range theory.

## Methods

### A realist evaluation case study

Realist evaluation is an approach within theory-driven evaluation [29]. Theory-driven evaluation was an important dimension of the discipline of evaluation during the 1980 s. Chen & Rossi developed it as a response to policy and programme evaluation approaches *that remain limited to before-after and input-output designs or *that focused too narrowly on methodological issues (method-driven evaluation) [30].

Realist evaluation proposes conceptual tools to apply the principles of theory-based evaluation and specifically aims at understanding the underlying mechanism between intervention and outcome. It aims at answering the question: 'Why does this work in this context and for whom?' rather than merely the question 'Does it work?'. The starting point of realist evaluation studies is a middle range theory (MRT) that is tested empirically and modified in accordance with the findings of each case. Middle-range theories should be understood as part of the theories of the middle range, defined by Merton as [31]: the "*theories that lie between the minor but necessary working hypotheses (...) and the all-inclusive systematic efforts to develop a unified theory that will explain all the observed uniformities of social behavior, social organization and social change*".

In practice, realist evaluators seek to analyse the case in terms of context-mechanism-outcome configurations, which offer an explanation of how the intervention led to the observed outcomes by describing the underlying mechanisms of change and the influence of the context on implementation and outcome. A realist study ends by adapting the initial middle range theory accordingly. This modified MRT then serves as a new hypothesis of the next study. This cycle refines the MRT and leads to better insights of how particular interventions work, in which conditions and how. Both qualitative and quantitative methods are used in the framework of case studies, which are linked to each other through the middle-range theory. We previously described the development and results of a realist case study of the influence of management on performance elsewhere [28].

The middle range theory that was tested in this case is the result of a literature review of the effect of high commitment management on health care performance [27] and of the concept of commitment, combined with previous empirical work in Ghana [28]. It may be summarised as follows:

In well-performing hospitals, the managers are driven by a strong vision and are capable of sharing this vision with their team and the staff at large.

Managers also work within the institutional arrangements that spell out their responsibilities and provide (access to) resources, but tend to utilise their decision spaces optimally.

In the management of the workforce, effective management teams combine administrative and commitment-eliciting management practices and adapt the mix according to the cadre, problem and task. They have a contingency view on HRM: they balance the configuration of management vision, management practices, the organisational tasks and staff, and organisational climate and culture.

A balanced HRM strategy consists of a combination of personnel administration (administrative HRM) and commitment-eliciting management. Such balanced bundles of management practices include goal setting, role distribution and task monitoring (structure) on one hand, and training, support and recognition (eliciting commitment) on the other.

Such a balanced bundle (through triggering perceived organisational support and reciprocity) leads to HRM outcomes like a positive organisational climate that stresses recognition, respect, commitment and trust.

Enduring effects of such practices can be expected if the organisational culture stresses the latter outcomes and professional values. To be an important determinant of performance, organisational culture requires an appropriate formal organisational structure.

Such balanced approaches assume a capacity for self-regulation, based on professionalism and public service ethos. Such balanced approaches demand time and a reasonable management capacity in terms of staff and competences.

### Study design

We used the case study design, as it allows exploring a "phenomenon within its real-life context, especially when the boundaries between phenomenon and context are not clearly evident" [32]. Furthermore, this design allows a holistic in-depth investigation of these issues as they happen in their natural setting, whereby different sources of information and data collection methods can be used concurrently [33]. A realist evaluation examines the generative mechanisms that underlie an intervention at micro- and macro-level, and explores how actors' choices and use of their resources led to the outcome. It also focuses on the embeddedness of the intervention in the social reality [29]. The case study design fits this bill nicely.

### Ethics

We assessed the ethical issues on the basis of the working paper "Notes regarding ethical guidelines for health services research" of the Department of Public Health, Institute of Tropical Medicine, Antwerp. This covered the following issues: Minimal risk to participants; Invitation, information and informed consent; Feedback to interviewees and staff. The protocol stipulated how potential interviewees would be (1) invited by letter, (2) given all relevant information about the study, (3) specifically informed before and after the interview of the possibility to opt out at any time, and (4) informed of the measures taken to protect anonymity and confidentiality. This study was presented for clearance by the hospital administration of the hospital and subsequently permission was granted.

### The case

We define the case as Ho Municipal Hospital (HMH), a Ghana Health Service Hospital (public hospital) that was commissioned in 1927. It is located in Ho, the capital town of Volta Region. In time, it became the regional hospital for the Volta Region. No major infrastructural works had been carried out since the 1950 s. Since the 1990 s, chronic under-funding of maintenance and repairs resulted in gradual deterioration of the infrastructure. In 1999, a new Volta Regional Hospital was commissioned and half of the staff of HMH was transferred to the new hospital. The old hospital was re-designated as the district hospital for Ho Municipality and Adaklu-Anyigbe District, with 150 beds serving an estimated population of 265,046 inhabitants (estimation on basis of census of 2000 [34]). In 2000, the outpatient clinics recorded 35.865 consultations per year and 7.034 patients were hospitalised. At this time, the hospital would have on average 2 Ghanaian general doctors, 1 Cuban specialist and 1 Cuban generalist and around 70 professional nurses for a bed capacity of 150. Of the remaining staff, only a third had post-secondary school qualifications. The Ministry of Health funding provided on average 25% of the hospital's recurrent budget, besides covering the wage bill. The remainder came from user fees for drugs, outpatient visits and hospitalisations [35].

The opening of the new regional hospital led to a drop in patient attendance at the municipal hospital and the staff's morale reportedly also plummeted. One ward was closed while major equipment fell into a state of disrepair [36]. The OPD attendance and the hospitalisations dropped continuously reaching their lowest point in 2004-2005. In 2006, multiple transfers led to the constitution of a virtually new management team. A new medical superintendent (the hospital director) was posted in April 2006. The accountant and the pharmacist were replaced, too. Finally, a new health services administrator took up his position in September 2006. Of the old team, only the nursing manager remained at post. After 2006, OPD attendance an admission rates increased steadily, despite a strike that paralysed services in June 2006. In addition, the volume of deliveries and of minor as well as some major operations increased. Following years of serious difficulties, Ho Municipal Hospital seemed finally to be on a rebound.

### Data collection

The quantitative part focused on data needed to describe the performance of the hospital. We drew data from the routine information system's records to produce overviews of trends in service production, revenue generation, expenditure, and staffing. The qualitative data was collected through document reviews, interviews and observations. First, a document review was undertaken of hospital records (annual reports and staffing reports), government documents (policy papers, HR statistics and reports), and publications. Second, the daily activities of certain wards, departments, and management meetings were observed. Third, interviews were carried out with a wide range of staff. These included semi-structured individual and group discussions with key informants to obtain information about their perception of the (changes in) management practices at the hospital, the organisational climate and the issue of organisational commitment. Two categories of staff were interviewed: (1) the 'operational staff' and (2) the management team members. We selected a stratified purposive sample that aimed at including staff members of various backgrounds.

• We first interviewed all 5 members of the core management team (average duration 58 min.) and 15 operational staff members, including nurses and nursing officers, doctors, para-medical staff, administration and support personnel (average duration 46 min, ranging from 27 min. to 1 h12 m).

• We undertook 6 group discussions to probe issues of organisational climate. The groups consisted of staff working in the same department, ranging from 3 to 8 staff members per discussion. Some consisted of members of the same cadre (for instance midwives working at the antenatal clinic), others included staff working in the same department (for instance theatre or laboratory staff). In total, 31 staff members participated in a group discussion, which on average took 42 minutes.

• Finally, we interviewed two health service managers from outside the district hospital to explore their view on Ho Municipal Hospital and the context issues of decentralisation, Ghana Health Service performance contracts, etc. (average duration 48 min).

All except 2 interviews were recorded and transcribed verbatim. After each interview, contact summary sheets were written, and field notes were written up at the end of the day.

The analysis started with the drafting of a thick description [37] and a timeline of events on the basis of a review of hospital records and reports. It mapped key internal and external events in time and was complemented by the interviews. Each research question was answered by exploring the relevant data sets. We used NVIVO 7 software for qualitative data management and analysis. The initial round of coding was based on the MRT and the research questions, from which a preliminary list of codes was drawn. This list was revised in the course of the interviews and the analysis. In a second round of analysis, we organised the emerging themes and patterns, guided by the categories from theory-driven evaluation [38]. We described the *intervention *(in this case the change strategy) in terms of content and application, and intended and actual outcomes. We drew on our interviews and observations to differentiate (proclaimed) vision (what the team wants), the discourse (what they say) and the actual practices (what they do). We described the organisational climate by analysing the interviews of the operational staff. In order to indicate how the intervention works, we identified and analysed both the *context *and the *intervening mechanisms*, and attempted to identify the essential *conditions*.

## Results

In this section, we describe the core management team's (CMT) intervention and its results in terms of organisational climate and health service provision (availability of services, volume and quality of services).

### The Core Management Team's intervention

**Figure 1:**
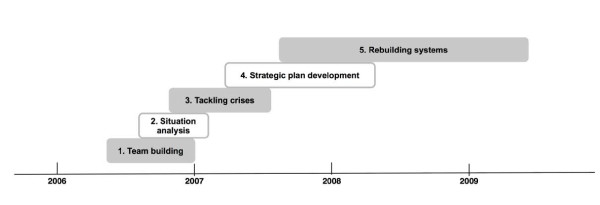
**Sequence of change management steps**.

Analysis of the documents and interviews indicates that the hospital went broadly through five phases since 2006. As shown by Figure 1, phase 1 (building the core management team), phase 2 (situation analysis) and phase 3 (tackling the crises) took place in 2006-07, while the development of the strategic plan was undertaken in 2007-08. In parallel, the main thrust of phase 5 (rebuilding the management systems) occurred in 2007-09.

### Phase 1 - Team building and the crafting of a shared vision

The first step consisted of constituting the core management team and the development of a shared vision on leadership and management. In April 2006, the M/Supt. was posted to the hospital. By September 2006, the arrival of the health services administrator made the team complete. In addition to the M/Supt., the team consisted of the nursing manager, the finance manager, the administrator and the pharmacist. This team initially met daily, and later on twice weekly.

Our interviews indicate that this core management team held regular meetings and brainstorming sessions, during which an initial plan to deal with the problems emerged. This consisted of re-launching the hospital through improving the motivation of the staff, rehabilitating the basic infrastructure and increasing the revenue of the hospital. The interviews indicate that the values and principles upon which this plan was based include: "excellence through self-reliance", "enhancement of problem solving capacity of staff", "best practices", "involvement and participation of staff and external stakeholders" and "delegation to the middle cadre".

Key elements of this leadership view include the central role of the staff themselves in shaping and maintaining change and in the subsequent choice of participative processes to induce change. Indeed, the team believed that the health workers at the hospital were key to their success. The implicit aim was to build upon the internal capacity and action of the staff:

So, you want to let [the staff] feel part of the circle, that everybody is within the circle, that you are necessary for whatever we are doing here. And that nobody from outside is coming to do this for us. So, when you look at our strategic plan, you realise that the theme is 'Rising internal strength'. In fact, this thing, we sat down and I could just do whatever and write the entire thing, but you make sure that everybody who came to the meeting contributes. So, everybody went through in participation so that you buy into whatever we are trying to put in place. (HMH IM 1-4)

The CMT felt that the development of the strategies to achieve this vision needed to be participative in nature and that workshops being held with all cadres were a good tool.

You see, everybody had to come on board, so workshops were held to really appeal to the people to change their attitude, depart from the old ways of doing things, because management was on the warpath. We requested to turn things around because management alone could not do that. Everybody had to come on board to make the hospital be at where it should be. (HMH IM 1-1)

### Phase 2 - A participative situation analysis

One of the first actions of the new management team was a situation analysis by means of a SWOT questionnaire distributed among all staff. The interviews show that this staff survey helped the management team members to know better their staff and their concerns. They picked up the general sense of frustration and were struck by the high number of elderly nurses and by the relatively high degree of illiteracy among lower cadres. The survey was followed by individual interviews with staff members of all cadres.

The team arrived at the following analysis: poor staff attitudes led to low quality of care and services, which was not accepted by the community. People stayed away and the subsequent weak revenue generation tied the hands of the management team. Inadequate maintenance further strained the already poor infrastructure. This all resulted in an organisational culture of fatalism, not countered by the previous management team. The results of this initial 'diagnosis' were presented and discussed during general staff meetings (durbars) at the end of 2006. This assessment was widely shared by the staff at that time.

Yes, this hospital was dying, almost dying, because it came to a point we even had to close down the theatre. We could not operate. That time, the attitude of staff towards the patients was not good. Doctors too were not there. So, for OPD, you will see less than 50 patients a day. [...] It came to a point, because patients were not happy with our services here, they decided to go to the Regional Hospital and Korle Bu, where they think they can be treated better. So, attendance here fell drastically before the new management came. (HMH GO 1-1a)

### Phase 3 - Tackling the crises

The CMT quickly initiated action to deal with the most urgent needs: the poor staff attitude, the low revenue generation, the low engagement of the mid-level cadre and the weak core administrative functions.

Improving the *staff attitude and communication *towards the patients was at the top of the agenda. The CMT carried out 2 patient satisfaction surveys in 2006, the results of which were used to sensitize the staff to the need for change. Subsequently, a workshop on "Effective Communication in Customer Care Service" was organised for all staff members. Ward conferences were organised to enhance the capacity of operational staff to analyse their problems and find their own solutions. The health information officer was actively involved in preparing ward-specific performance analyses and the patient and staff satisfaction surveys. The resulting information was fed back to the units to stimulate improvement.

A second priority was the *rehabilitation of prime resource generating service points*. Repairs were carried out at the operation theatre in 2006, and major operations started again. In July 2006, the National Health Insurance Scheme (NHIS) temporarily accredited the hospital. The M/Supt. initiated a registration drive in the district, together with the district NHIS office. At the end of the year, the proportion of patients registered with the NHIS had risen to 13,1%. Subsequently, the hospital began the rehabilitation of the mortuary, for which the hospital staff agreed to lend funds from their Staff Welfare Funds. The mortuary commenced operations in 2007. Successful efforts were also made to keep up the stock of drugs.

Third, the CMT revived the *regular communication channels *with the aim to involve the mid-level managers and to better stream information up and down the organisation. The regular committees, which are supposed to operate in all GHS institutions, were a key element. The monthly Heads of Unit Meeting, uniting the heads of all units and departments of the hospital, was reinstated, as well as the procurement committee and the staff durbars. The Hospital Management Committee, more restricted in membership than the Heads of Unit meeting and providing strategic guidance to the CMT, was revived. For strategic reasons, all doctors as well as some external actors like the Ho Municipal District Director and the Regional Public Health Nurse were included in the Hospital Management Committee. This committee subsequently met monthly. Also, contact with key external stakeholders was re-established, including the district National Health Insurance Scheme office, the Regional Health Directorate and the Municipal District Health Director. Relations with the local chief and with the press were taken care of. A 'Hospital Open Day' was organised to enhance involvement of the community and other stakeholders and to start disseminating the message of change.

Finally, measures were taken to improve *core administrative functions *needed to ensure improved revenue collection. NHIS claim processing was streamlined and additional accountants were hired.

### Phase 4 - Development and consolidation of the 5 year strategic plan

In 2007, the CMT felt that the dynamics of change would be better sustained by a strategic plan for the hospital. Its development was initiated with the hiring of two lecturers of the Unit of Change Management, Ho Polytechnic. A staff member of the Regional Health Directorate assisted this team. The initial process consisted of a participative SWOT analysis and environmental scanning, followed by a cycle of focus group discussions with staff from all cadres. On this basis, the Polytechnic team drafted a preliminary plan. The CMT revised the mission statement and the action plan at the end of 2007. The resulting Strategic Plan 2008-2013 "Rising [sic] internal strength" [39] was consolidated through extensive consultation with the staff and external stakeholders, including representatives of NGOs, the Municipal Health Directorate and the District Assembly.

The first part of the plan presents a synthesis of the SWOT analysis, the basic assumptions and forecasts on which the plan is based, and the vision, mission, core values and objectives of the hospital (see Table 1). The second part presents the main strategies and detailed action plans [39]. The plan was launched officially in January 2008, in the presence of a number of MOH and GHS senior staff. T-shirts, pamphlets, flyers, copies of the strategic plan and meeting calendars were widely distributed: a deliberate strategy to create visible signs of change. More importantly perhaps, a new information channel was created: the monthly hospital information bulletin targets staff and external actors and disseminates information on management interventions and on the performance of the departments.

**Table 1:** Key elements from the Strategic Plan 2008-2014 "Rising internal strength"

The core values	Patient/client at the centre, irrespective of status
	Fair treatment to all
	Team spirit and harmony at all levels to enhance synergy
	Efficient utilisation of resources to improve accountability
	Rationalisation through dissemination of best practices
	To be sensitive to the environment
The main strategies	To improve the quality of care
	To strengthen the hospital's health workforce
	To ensure adequate infrastructure
	To increase revenue
	To improve the management systems

### Phase 5 - Rebuilding the systems

**Figure 2:**
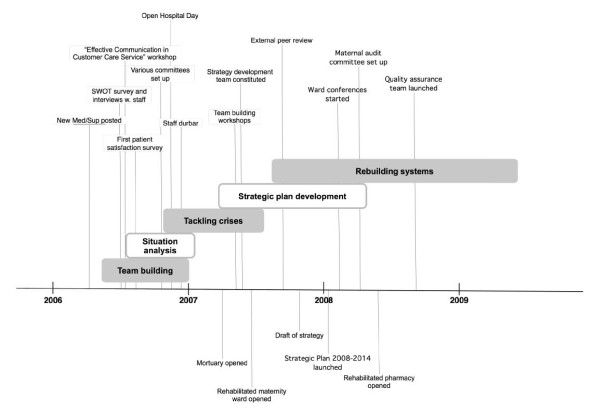
**Sequence of management steps and outcomes**.

While the CMT tackled weaknesses in some administrative and management systems early on (see phase 3), the strategic plan 'Rising the internal strength' presented a long-term vision to improve the clinical quality assurance systems, administrative functions, and management of infrastructure and logistics. Figure 2 presents a timeline of the main interventions and managerial outcomes.

First, several initiatives were taken to institutionalise quality assurance. A series of training workshops in the use of the GHS quality assurance manual was organised for all staff, and special ward conferences addressed particular issues. In 2008, the maternal mortality audit committee was re-launched and maternal mortality audits organised. Observational studies of waiting time at the OPD and the patient satisfaction survey led to a workshop for OPD staff during which the patient flow was reorganised with the aim to reduce waiting time and improving privacy.

The data collection, analysis and use of routine health information were improved. For instance, when the Health Information Office picked up a sharp increase in stillbirths in 2007, a training workshop in resuscitation skills was organised in response. The laboratory set up formal quality assurance systems. At the pharmacy, prescriber behaviour was analysed and discussed with the providers. In 2007, the CMT organized an external review by a peer visitation team, a first in the Volta Region. During this peer review of the Hospital, health professionals from other hospitals in the Volta Region visit one hospital and review all its activities using a scoring system. Management and operational systems are reviewed through observation and review of functions and practices.

Second, several administrative and management systems were addressed, starting with the strategic planning capacity by the very development of the strategic plan. Financial management was strengthened by hiring additional accountants. Procurement of drugs was streamlined.

Third, after the emergency repairs in 2006, major rehabilitation works started in 2007, leading to the opening of the rehabilitated maternity ward in 2007, and of the pharmacy, laboratory and the offices of the Health Information System (HIS) department in 2008. At the outpatient department, the roofing of the building was reconstructed and an extension was made to the structure. A start was made with the installation of networked computers at every service point, the accounting and HIS offices.

### The organisational climate

The organisational climate is defined as the atmosphere that employees perceive, which in turn is created in their organisation by management practices, procedures and rewards. In other words, it reflects how the staff feel about their relations with their managers and their perceptions regarding the interventions of the management team. Four particular themes emerged during the analysis: the perceptions about changed leadership, the intervention of the CMT as perceived by the operational staff, perceptions of positive change and perceptions of support for the workforce as well the revamped institution.

### The perception of changed leadership

The changes in leadership were perceived as an important event by most interviewees. They describe their relations with the CMT members, their approach to communication and their accessibility and note the effects on staff morale.

In those days, the relationship was not cordial. That time, the way the former management handled staff was not the best. So, some of the staff were discouraged from rendering their services because of the way things were happening, but now, everybody is trying to do his best because of the way they handle us, because there is a change. (HMH GO 1-4)

The interviewees noted especially the new vision on leadership and management and specifically the emphasis on participation. For instance, interviewees appreciated the efforts to ensure a participative process that would define the hospital's mission and the core values, or to the problem analysis exercise in which they were also involved. They note how this participative approach was applied at the management level of the hospital and at the interface with the operational staff. For the former, the interviews show that the strategy was to create a critical mass of staff supporting the changes. Indeed, the M/Supt. delegated responsibilities to take initiatives and to implement the change agenda to a group of staff members committed to change. The ward conferences reconnected the CMT to the mid-level cadre and the operational staff. Later, these ward conferences and the quality assurance workshops were used to maintain the drive of the plan and the organisational vision.

We want to communicate with the staff, we want to build their confidence, we want them to take and participate in whatever that is happening. And one way to do this is the ward conference. Above all, we want to teach them methods of arriving at certain things with that method. How do you arrive at problem solving? This is a problem, so in the ward let's sit down, what do you do in other to do this and that. (HMH IM 1-4)

Many interviewees recognise the role of the M/Supt. in making this a truly shared vision on leadership and management, and his emphasis on teamwork and information sharing.

In fact, the medical superintendent is a key player in the team. He inspires us. He works with us as a team. Even if he is having the idea, he calls us, discusses it with us and we also make our contributions and whatever is agreed on, then we start implementing. So, it is like, all the time, we are all together in the work. Even if he has taken a decision which needed to be taken instantly he calls us and tell us this is why he has done this and that. So, we are not left out of the game. (HMH IM 1-5)

But for the key ones who understand, we will even come to you and tell you: "We are doing this and that, why can't we do this other thing". Sometimes you may not necessarily go to them but they may come to you with what they're thinking. [...] So, that is how we discovered them and that is how we try to include them in whatever, so that they don't get frustrated and they keep on thinking about what is at stake. (HMH IM 1-6)

This inclusive approach, however, met with some resistance, both from operational staff (for instance the theatre staff) as well as from management team members, who objected when the M/Supt. wanted junior staff members to join the CMT. The director finally gave in to the CMT, but kept the junior staff in the loop by delegating key tasks to them, which served to keep them committed to improving the hospital.

### How the management intervention is perceived by operational staff

When asked about the key interventions of the CMT, the interviewees refer commonly to 4 main elements: delegation to the units, improved flow of information through the committee system, team work and shared values.

First, the interviewees recognise that the CMT facilitates the delegation of problem analysis and - solving to the departments, a main aim of the 5-year strategic plan.

You know, [the CMT] doesn't want to impose things on us. [...] What I mean is that there are things that we ourselves can do. There are some problems, which everyone among us can solve. Do we wait for management to come and solve those problems? So, they want to draw our attention to those things. It is not everything that you sit down for management to come and do it for you. No, we must try as much as possible to do it ourselves. (HMH GO 1-2)

Second, the interviewed health care providers express the feeling that this delegation is effectively accompanied by a better information flow through staff durbars, the monthly bulletin, posters and reports. Especially the management meetings and the follow-up of issues are perceived as positive. The ward conferences are appreciated because they are oriented towards problem analysis and effective solutions. The participative nature of the strategy development and implementation seems to have led to a sense of involvement.

The strategic plan was developed in consultation of some experts from outside. They came and consulted with a lot of the units, deliberated, took our recommendations and they made their findings to the management. Then, later on, they came back to the various units, spelled out the findings, whether it's really good for the various units. Then we made our adjustments and input to those ones. Then, finally, they send it to the experts to refine it until it became a document. So, there was interaction until the final document came out. (HMH IO 1-7)

Third, the diversity of interacting committees, from the unit meeting, over the head of units meeting and the hospital management team to the core management meeting all contributed to a positive sharing of information between 'bottom' and 'top' of the hospital, but also to a sense of being part of the team (inclusion) and a perception of sharing values among staff.

This management has really brought that much. They have always done this to show that we are part of the process. We are in one car moving in one direction. Very often they bring some forms for us to fill: how do you feel about the hospital, how do you feel about your leader, how do you feel about this worker. We always have such interactions. So, I think we have very positive mind of the management so far. (HMH IO 1-8)

Finally, the broad involvement of staff in the workshops and ward conferences has led to better teamwork and collaboration between staff of various units, and strengthened the sharing of the values of the hospital, especially that of 'working for the patient'.

I feel there is a change. And I think in terms of communication too, the patient and workers relationship has improved and that was as a result of seminars that were organised. (HMH GO 1-4)

Love. We have been told by the authorities to really do it for love and that thing has been preached so many times, to do it for love. That is what is keeping most of us moving with the hope that one day it would be better. [...] People are now working with their heart. They are working not because of what they are getting, but they are working because of a certain satisfaction. Like one nurse said, when I nurse my patient from a very bad situation of sickness to a better one, then it's a joy. (HMH IO 1-2)

### Perception of positive change: some mixed feelings

The interviews show that the workshops and initial wave of improvements of the hospital infrastructure lifted the spirits of many staff members.

As for now, things are improving. Look at our Maternity Wards over there, and the new signboards we are having here. The mortuary was broken down for so many years. Now the mortuary is working and it is bringing more money. And so now, we all feel proud and we hope very soon, they will rehabilitate the whole hospital. (HMH IO 1-6)

They state that the hospital gained again the trust of the population of Ho and that this explains the increase in the volume of patients.

I have seen a lot of change. Initially, client attendance was less when I came. People normally talk about the hospital, that if you go there the human relations are poor. That was one big problem. For instance the nurses, they insulted and they talked to you anyhow. There was no confidentiality, they shouted and people heard whatever was going on. (HMH IO 1-8)

However, the hospital personnel is in a sense, also the victim of its own success: the higher volumes of patients increase the workload and staff start claiming additional incentives. In general, the initial promises and successes seem to have raised high expectations, which cannot all be met given the resource constraints.

Now the pressure of work has increased, and now everyone wants motivation and when motivation is not coming... (HMH IO 1-5)

### Perception of support

Regarding the perception of support by management, some staff remained sceptical. In the group discussions, theatre staff expressed their dissatisfaction despite the repairs made in the theatre. Orderlies complained about poor supplies of cleaning equipment and consumables. Casual workers complained about receiving fewer benefits than staff on permanent contract.

Since I came here, the changes, in particular at theatre, have been negative, because things continue to deteriorate. When you enter the theatre now, the wall tiles are all falling down. The roofs are leaking. The windows of the theatre, which are supposed to be closed or airtight because of the theatre, are open. They cannot close well. [...] Our equipments are obsolete and we are not using them. So in short, things have been going negative. There is not much improvement. (HMH GO 1-1)

However, most of the operational staff members we interviewed feel that their problems are recognised and effectively solved by the management team. This support is felt through a number of actions of the CMT: the relationship between management team members and the staff, their work floor presence, the meetings and easy informal access to the CMT members. They express this support by the management team as 'keeping promises'.

Interviewees say that the good cordial relationship between management team members and the staff is important to them. This relationship is felt to be fair and supportive. It is also a factor that improves the motivation and retention of health workers at Ho Municipal Hospital.

Because I have established a nice relationship with management, I will feel better working here than going elsewhere with the same condition. Upon that, just because their facilities are better, no, no, I don't see it like that. I prefer working here, because the people you are working with have a good relationship with you and that makes you going and staying stronger. That is keeping us here, otherwise most of us here would have gone. The spirit of the people, our leaders, our colleagues around, they are supportive. (HMH IO 1-8)

Although the work floor presence of CMT members is variable - some turn round everyday, others rarely - it is perceived as a strong signal of interest and support.

As I have already said, they come round to see what you are doing and give you support appropriately. In that aspect, we are one. (HMH IO 1-5)

Also unit meetings were in general perceived as effectively channelling concerns, problems and information to the core management team about the operational units, facilitating effective support by the CMT. Besides these formal channels of interaction, several interviewees indicated that access to the CMT members is good: they could easily go and see management team members in case of problems that could not be solved with their supervisor. They perceive especially the M/Supt. as "*really listening to us*".

### Hospital performance

**Figure 3:**
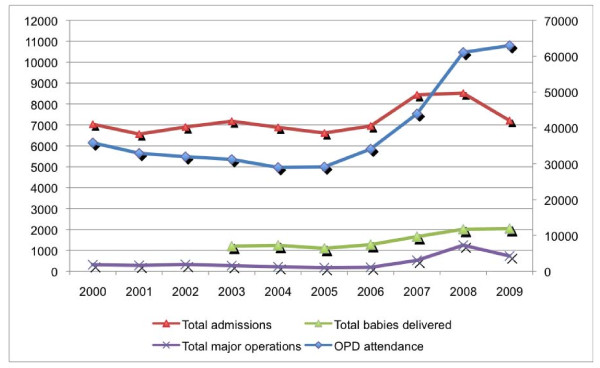
**Volume of services (OPD, admissions, maternity and theatre for Ho Municipal Hospital, 2000-2009)**.

As already mentioned in the introduction, the OPD attendance doubled to more than 62,000 consultations in 2009, from around 30,000 consultations per year during the period 2002-05. Also, the number of admissions increased, but less dramatically than the OPD attendance, from 6,882 admissions in 2004 to 8,512 admissions in 2008, dropping slightly in 2009. The admission rate gradually declined, from 37% in 2004 to 14% in 2008, a sign of more selective hospitalisation. However, since we could not assess the evolution of the case mix, firm conclusions cannot be drawn from these figures.. The total number of deliveries at HMH almost doubled in 5 years' increasing from 1,163 in 2003 to more than 2,000 deliveries in 2009. Data on Caesarean sections however could not be fully retrieved (see Figure 3).

An important change in the context was the start of the National Health Insurance Scheme in 2006, which stimulated the utilisation of health services in Ghana.

In Ho Municipal District, the OPD attendance at all facilities combined has increased from 0.45 new cases/inhabitant/year in 2005 to 0.8 in 2008. This supports the idea that the NHIS increased access to virtually all health facilities in the district. However, this increase of utilisation was not uniform for all facilities. Indeed, the Ho Policlinic (a Ghana Health Service facility) saw a decrease in utilisation between 2004 and 2007. Only in 2008 was there an increased attendance. At the Volta Regional Hospital (another Ghana Health Service facility in Ho town), the OPD attendance has been decreasing since 2005. Here, it could be argued that the gate-keeping system may have contributed to this decrease: regional hospitals were not supposed to accept patients who could be dealt with at lower-tier health facilities. In practice, it may have done so, but only in 2006 and in the first half of 2007. Since then, non-referred patients have been admitted to the regional hospital's OPD, and it even set up a primary care service to attract patients.

Management of clinical care quality is reflected by hospital mortality rates. While the mortality increased in absolute numbers, in line with the higher number of admissions, the crude mortality rate (N of deaths/1,000 admissions) remained around 45/1,000 admissions since 2004.

Tracer drug availability has increased steadily since 2005, despite the increasing number of out- and in-patients. However, regarding the availability of consumables, interviewees indicate that disinfectants, gloves, needles and syringes are regularly out of stock.

Revenue generation at HMH has dramatically increased over the last 4 years. Actual expenditure has kept up well with the revenue generated and mobilised, with expenditure rates above 90% except in 2006, when only 82% of the actual revenue was spent (Table 2).

**Table 2:** Revenue generation and expenditure at HMH, 2000-09 (Source annual reports and accounting records HMH)

	2000	2001	2002	2003	2004	2005	2006	2007	2008	2009
Total revenue generated (Ghana Cedi)	166,654	158,581	195,497	249,748	298,853	317,709	377,973	730,670	1,316,638	1,387,883

Total expenditure (Ghana Cedi)	148,491	140,738	200,509	228,597	276,615	309,531	310,469	656,643	1,186,927	1,389,009

% of revenue spent	89.1	88.7	102.6	91.5	92.6	97.4	82.1	89.9	90.1	100.1

Note: Exchange rate: 100 Ghana Cedis = approximately US$ 70

## Summary

We found that the management team combined a crisis management plan with the development of a long-term strategy (*the intervention*). On the basis of their shared vision of what the hospital should become and on what good leadership should be, it set out to mobilise and motivate their workforce (*intended proximal outcome*) by involving staff in both the diagnosis and the search for solutions (*expected mechanism*). At the same time, they strengthened the management structures (ensuring a conducive work environment and streamlining the internal administrative organisation and procedures) (*expected mechanism*). This combination was intended to lead to better performance of the hospital (*intended distant outcome*).

We saw that the first initiatives taken by the core management team - investing in staff, communication, revenue generating capacity and infrastructure - lifted the spirits of staff and that this led to a change in attitudes of staff towards patients (*observed actual proximal outcome*).

We also found that participation and inclusion, improved information sharing and communication between director, management team and staff, in addition to the visible improvements, led to a strong sense of being supported amongst most staff (perceived organisational support), and to a positive organisational climate (*mechanisms*). For some cadres, however, the change and support is not sufficient (*failure of mechanism for some staff cadres) *and they feel clearly frustrated *(unequal distribution of intermediate outcome*).

We also note an increase in most performance indicators. The volume of patients (OPD, hospitalisations, maternity) improved, as well as the revenue (*measured actual distant outcomes*). However, this cannot solely be attributed to the management changes, since utilisation of health care services increased all over Ghana since the start of the NHIS *(implementation context*). However, HMH improved its utilisation more than the other facilities in Ho, which all can draw on NHIS reimbursements. This may indicate that other factors besides the NHIS funding contributed to increased utilisation at HMH - plausibly the management approach.

## Analysis

In a realist case study, the analysis consists of searching for patterns in the form of context-mechanism-outcome (CMO) configurations, whereby the analyst is guided but not constrained by the key elements of the initial middle range theory. In our case, the initial key elements included: leadership, creation of a shared vision, the choice of management practices, mechanisms of reciprocity and outcomes at levels of HRM and hospital performance (see Methods).

We found during the analysis that the patterns centred on the themes of participation, empowerment and reciprocity.

The first theme is 'participation', reflected by the joint problem analysis, construction of the mission and strategic plan for the hospital, and in the organisation of workshops and ward conferences. Increased participation of operational staff resulted in reconnecting a fragmented organisation. By reviving the regular committees and initiating the ward conferences, the CMT reconnected the operational staff to their unit heads, and the unit heads to the CMT. The system of inclusive workshops gave all staff the possibility to engage in hospital affairs. As we've described, major efforts were made to delegate decision making to the operational units and a number of committees. This cascade of interlocking committees channels up and enables information and concerns from the operational units to the central levels of decision-making.

The striking feature of the unit meeting is that before the Unit Heads come for Unit Heads meeting, they must have held in-house unit meeting at the unit level to equip the unit manager with the problems militating against his unit before he even appears at the Unit Heads meeting. So he comes armed with the collective decision from the Unit. So that whatever he says there he speaks for the unit. (HMH IM 1-1)

Another example of how participation works is the 'ward conference', whereby the situation of a particular unit is analysed by its staff, supported by a few CMT members. We found these conferences to be an effective way of decentralising decision-making power to the operational level. They are perceived as useful by operational staff members, not only because they allow information to be passed up and down the hierarchy, but also because staff problems are discussed and solutions sought. Others point out how these meetings involve and engage staff at the operational level.

What they do is organizing these seminars and the rest. It is one way of doing that; if you bring the workers closer to you, then you are going to tap their ideas/views and it makes people feel good because there are people who may equally contribute to the progress of the hospital but if given the chance. So, these seminars and workshops have really given them the platform to really make their point and it is a way of bringing out our grievances. You know, dealing with grievances is one aspect, which is so dear to workers. If you as a manager don't know the in and out of your workers, solving such grievances becomes difficult; you get to know the why and how things happen; and since they are closer to us it is easier for them to correct you where you are going astray. (HMH IO 1-8)

By stressing participation, the CMT stimulated the sense of self-capacity of many interviewees (empowerment). As one of the staff actively involved in the organisation of the workshops mentions:

Most of the time, if the M/Supt. realises that you have the potential to work, he tries to delegate some of his responsibilities. [...] How he is doing it is that he doesn't just look within the core management, but he looks beyond the core management. Those who have the capacity and those who share his vision, he tries to bring them in. Some of us have been doing that for him. (HMH IO 1-13)

Indeed, for a significant number of staff, the call of the CMT for their involvement was a sign of recognition. This resulted in feelings of reciprocity, but not among all: expectations may have been too high for some. To some extent, the orderlies are up against a difficult task: given the poor infrastructure, the needs for cleaning are great and the resources still too limited. The theatre staff is clearly frustrated despite the improvements made in infrastructure and supplies. It may well be that there are deeper underlying conflicts that may explain these staff's unease with the CMT.

It should be noted, however, that this commitment-eliciting approach was combined with a strategy to strengthen the administrative management systems and operational support in terms of supplies and facilitating working environment (for instance in outpatient department, laboratory, radiology and the health information unit).

The negative reactions of some cadres indicate that the initial change of climate, from poor staff attitudes to a sense of being capable and of being proud to work at HMH, may prove difficult to maintain, if it is not followed by permanent reinforcing mechanisms and by induction procedures for new staff.

Conditions that enabled this approach at HMH include the bad condition in which the hospital was - there were few options other than to change dramatically - and the strong support from the Regional Health Directorate. Also, the NHIS came at the right moment and the opportunities it provided were quickly grasped by the CMT.

## Discussion

It could be argued that the limits of this study include the traditional limitations of the case study design in terms of internal and external validity, whereby the findings reflect to some extent the researchers' perspective and may only be relevant for the local context of the study site. We would argue that the theory-driven approach however addresses both issues by forcing the researcher to make explicit the initial hypothesis in the form of a MRT that describes the assumed mechanisms and the influence of the context. This explicit MRT is the framework that guides the analysis, with the explicit aim to refine it by confirming or refuting its elements on the basis of the analysis of empirical findings. It also forces the researchers to identify the key conditions for this intervention to work in this context.

In Pawson and Tilley's view, a realist evaluator does not strive nor pretends to provide the ultimate evidence that the intervention works, but rather to enlighten the decision-maker by providing plausible explanations. The structured description of intervention, outcomes at different levels, context conditions and mechanisms allows the reader to judge whether this explanation is plausible and whether such intervention, by triggering specific mechanisms, may have similar effects in other environments. We refer to an earlier publication for a more detailed discussion on the methodological issues of doing a realist evaluation [28].

A difficult question is the attribution of the increased utilisation and revenue of the hospital. Without doubt, the rapid expansion of the National Health Insurance Scheme contributed to a substantial increase in utilisation rates at HMH, as it did in the whole of Ghana. However, the revenue generated from providing services to non-NHIS members also increased. Furthermore, HMH did noticeably better than other health facilities in the town of Ho, including the regional hospital. We argue therefore that the introduction of new management practices by the new CMT plausibly explain the U turn.

### Adapting the MRT

Our initial MRT - the result of previous case studies and literature reviews - states that "in the management of health workers, effective management teams combine administrative and commitment-eliciting management practices and adapt the mix in function of cadre, problem and task. They have a contingency view on HRM: they balance the configuration of management vision, management practices, the organisational tasks and staff, and organisational climate and culture." It also stipulates that both a commitment-eliciting approach and a structuring approach need to be combined. We found that this indeed mattered in HMH. The results of our case study at Ho confirm the main lines of this initial MRT, even if the setting was one of a crisis.

There are, however, new elements. Regarding the mechanisms, this case points to the influence of leadership vision on the effect of HiCom practices. *Inclusion*, *participation*, *involvement *and *empowerment *were key attributes of the management intervention and led to a positive organisational climate and to the perception of *organisational support*. Indeed, the strategy of trying to include all staff in the dynamics of change, through mobilising a critical mass of close collaborators and through workshops for all operational staff, has contributed to a feeling among most staff of being effectively supported by management. This perceived organisational support has been shown to lead to higher organisational commitment through the mechanism of reciprocity [40,41]. We would argue that a leadership style that facilitates inclusion and participation triggers commitment and reciprocity through the mechanism of perceived organisational support.

In summary, the results of this case allow us to modify the initial MRT as follows (see text in italics)

In well-performing hospitals, the managers are driven by a strong vision and are capable of sharing this vision with their team and the staff at large.

Managers also work within the institutional arrangements that spell out their responsibilities and provide (access to) resources, but tend to utilise their decision spaces optimally.

In the management of health workers, effective management teams combine administrative and commitment-eliciting management practices and adapt the mix according to the cadre, problem and task. They have a contingency view on HRM: they balance the configuration of management vision, management practices, the organisational tasks and staff, and organisational climate and culture.

A balanced HRM strategy consists of a combination of personnel administration (administrative HRM) and commitment-eliciting management. Such balanced bundles of management practices include goal setting, role distribution and task monitoring (structure) on one hand, and training, support and recognition (eliciting commitment) on the other. *These practices need, however, to be responsive to the different cadres. This adaptation requires permanent good communication with all staff*.

Triggering perceived organisational support and reciprocity, a balanced bundle leads to HRM outcomes like a positive organisational climate that features *inclusion*, recognition, respect, commitment and trust.

Enduring effects of such practices can be expected if the organisational culture stresses the importance of participation, commitment and empowerment, and professional values. To be an important determinant of performance, organisational culture requires structure.

Such balanced approaches assume a capacity for self-regulation, for instance based on professionalism and public service ethos. Such balanced approaches demand time and thus a reasonable management capacity in terms of staff and competences.

### Putting the findings in perspective

Our findings correspond with the views of Alimo-Metcalfe and colleagues about engaging leadership. The style of leadership at Ho was decidedly aimed at increasing the engagement of staff (commitment-eliciting): the CMT did not aim at simply imposing their analysis and solutions to the middle-level cadre and operational staff, but adopted a participative and enabling style of leadership. This management vision had a positive influence on the implementation of their set of practices and on how the staff perceived these practices. This led in turn to a positive organisational climate in most departments and wards. Staff felt supported and in return were willing to go the extra mile for the hospital.

However, the CMT also initiated structural changes in the sense of strengthening the administrative, financial and procurement procedures and management systems. In that sense, they adopted rather a contingency approach to leadership and management, combining a hard and soft approach. This is in tune with research on high performance in primary health care organisations. Ohman-Strickland and colleagues show how a number of similar organisational attributes lead to better organisational performance [42]:

• Leadership: a focus on effective problem solving through engagement of stakeholders and sharing of information

• Organizational culture: stimulating openness, connectedness and learning

• Relationships: fostering collaboration and communication

• Management functions: clear structures and role distribution in the domains of financial management, logistics and infrastructure, healthcare provision, HRM and strategic planning

• Information mastery: access to and use of information for learning and problem solving

Finally, if the findings are framed in the change management perspective, they reflect many of the key conditions of success identified by Kotter: establishing a sense of urgency, setting up a coalition, developing a shared vision, strong communication, removing obstacles to change, planning systematically, creating short-term success and anchoring change in the organisational culture [43].

## Conclusions

Few studies on hospital management in LMIC have been published, despite a well-developed body of knowledge of how management can influence performance in the disciplines of human resource management and strategic management. This study shows that HiCom management is being implemented in a Ghanaian district hospital and that such an approach contributed to better performance. The hospital management team at Ho incorporated a balanced administrative and commitment-eliciting HR strategy within an overall change strategy. By triggering mechanisms of staff participation, empowerment and reciprocity, it instigated a U-turn in hospital performance. This case also shows how a realist evaluation approach can be used to study management interventions in complex settings such as a hospital. However, additional work is needed to explore the specific contextual factors that enable such balanced HRM practices to work, and how the dynamics of change could be institutionalised for sustained improvement in hospital management practice.

## Competing interests

The authors declare that they have no competing interests.

## Authors' contributions

All three authors contributed to the original design of the study. BM carried out the data collection. BM and MD analysed the data. BM, MD and GK contributed to the discussion section and to the writing of the manuscript. BM edited the final draft. All authors read and approved the final manuscript.

## Pre-publication history

The pre-publication history for this paper can be accessed here:

http://www.biomedcentral.com/1471-2458/10/787/prepub

## References

[B1] Chopra M Munro S Lavis JN Vist G Bennett S Effects of policy options for human resources for health: an analysis of systematic reviews Lancet 2008 371 9613 668 674 10.1016/S0140-6736(08)60305-0 18295024

[B2] Walton R From control to commitment in the workplace Harv Bus Rev 1985

[B3] Morgan D Zeffane R Employee involvement, organisational change and trust in management International Journal of Human Resource Management 2003 14 1 55 75 10.1080/09585190210158510

[B4] Evans W Davis W High-performance work systems and organisational performance: the mediating role of internal social structure Journal of Management 2005 31 5 758 775 10.1177/0149206305279370

[B5] Pfeffer J Veiga J Putting people first for organisational success The Academy of Management Executive 1999 13 2 37 48

[B6] Whitfield K Poole M Organizing employment for high performance: theories, evidence and policy Organization Studies 1997 18 5 745 764 10.1177/017084069701800502

[B7] Guest DE Human resource management: when research confronts theory International Journal of Human Resource Management 2001 12 7 1092 1106 10.1080/09585190110067837

[B8] Wright P Boswell W Desagregating HRM: a review and synthesis of micro and macro human resource management research Journal of Management 2002 28 3 247 276 10.1177/014920630202800302

[B9] Harris C Cortvriend P Hyde P Human resource management and performance in healthcare organisations J Health Organ Manag 2007 21 4-5 448 459 10.1108/14777260710778961 17933375

[B10] Alimo-Metcalfe B Alban-Metcalfe J Engaging leadership: creating organisations that maximise the potential of their people Research Insight 2008 London: Chartered Institute of Personnel and Development

[B11] Bradley M Alimo-Metcalfe B Leadership. Best actors in a supporting role Health Serv J 2008 28 29 18561422

[B12] Glisson C Assessing and changing organisational culture and climate for effective services Research on Social Work Practice 2007 17 6 736 747 10.1177/1049731507301659

[B13] Schneider B Gunnarson S Niles-Jolly K Creating the climate and culture of success Organisational Dynamics 2004 23 1 17 29 10.1016/0090-2616(94)90085-X

[B14] Brunet L Savoie A Le climat de travail 1999 Montréal: Editions Logiques

[B15] Podsakoff P MacKenzie S Beth Paine J Bachrach D Organisational citizenship behaviors: a critical review of the theoretical and empirical literature and suggestions for future research Journal of Management 2000 26 3 513 563 10.1177/014920630002600307

[B16] Schein E Organizational culture American Psychologist 1990 45 2 109 119 10.1037/0003-066X.45.2.109

[B17] Davies HT Mannion R Jacobs R Powell AE Marshall MN Exploring the relationship between senior management team culture and hospital performance Med Care Res Rev 2007 64 1 46 65 10.1177/1077558706296240 17213457

[B18] WHO Managing the health Millennium Development Goals - the challenge of management strengthening: lessons from three countries 2007 Geneva: World Health Organisation

[B19] Onzubo P Turnover of health professionals in the general hospitals in West Nile Region Health Policy and Development 2007 5 1 30 36

[B20] Couper I Hugo J Management of district hospitals - exploring success Rural and Remote Health 2005 5 433 16207080

[B21] Agyepong I Anafi P Asiamah E Ansah E Ashon D Narh-Domotey C Health worker (internal customer) satisfaction and motivation in the public sector in Ghana International Journal of Health Planning and Management 2004 19 319 336 10.1002/hpm.770 15688876

[B22] Mutizwa-Mangiza D The impact of health sector reform on public sector health worker motivation in Zimbabwe Bethesda: Partnerships for Health Reform 1998

[B23] Hagopian A Ofosu A Fatusi A Biritwum R Essel A Gary Hart L Watts C The flight of physicians from West Africa: Views of African physicians and implications for policy Soc Sci Med 2005 61 8 1750 1760 15927335 10.1016/j.socscimed.2005.03.027

[B24] Kipp W Kamugisha J Jacobs P Burnham G Rubaale T User fees, health staff incentives and service utilization in Kabarole District, Uganda Bulletin of the World Health Organization 2001 79 11 1032 1037 11731810 PMC2566686

[B25] Couper ID Hugo JF Tumbo JM Harvey BM Malete NH Key issues in clinic functioning - a case study of two clinics S Afr Med J 2007 97 2 124 129 17404674

[B26] Puoane T Cuming K Sanders D Ashworth A Why do some hospitals achieve better care of severely malnourished children than others? Five-year follow-up of rural hospitals in Eastern Cape, South Africa Health Policy Plan 2008 23 6 428 437 10.1093/heapol/czn036 18796499

[B27] Marchal B Kegels G Focusing on the software of managing health workers: What can we learn from high commitment management practices? International Journal of Health Planning and Management 2008 23 4 299 311 10.1002/hpm.882 17624868

[B28] Marchal B Dedzo M Kegels G A realist evaluation of the management of a well-performing regional hospital in Ghana BMC Health Services Research 2010 10 24 20100330 10.1186/1472-6963-10-24 PMC2828434

[B29] Pawson R Tilley N Realistic Evaluation 1997 London: Sage

[B30] Chen H-T Rossi P The theory-driven approach to validity Evaluation and Program Planning 1987 10 95 103 10.1016/0149-7189(87)90025-5

[B31] Merton RK Social theory and social structure 1968 New York: The Free Press

[B32] Yin R Case study research. Design and methods 2003 Third London: Sage Publications

[B33] Denscombe M The good research guide for small-scale social research projects 2003 2 Maidenhead: Open University Press

[B34] GHS Ho Municipal Hospital Annual Report 2006 2006 Ho: Ghana Health Service

[B35] HMH Annual Report Ho Municipal Hospital 2002 Ho, Ghana: Ho Municipal Hospital, Ghana Health Service

[B36] HMH HMH 2006 Performance appraisal 2006 Ho, Ghana: Ho Municipal Hospital, Ghana Health Service

[B37] Geertz C The interpretation of cultures 1973 New York: Basic Books

[B38] Chen H-T Theory-driven evaluations 1990 1 Newbury Park, California: Sage Publications

[B39] HMH Rising internal strength. 5-year strategic plan 2008-2013 - Ho Municipal Hospital 2008 Ho: Ho Municipal Hospital, Ghana Health Service

[B40] Eisenberger R Fasolo P Davis-laMastro V Perceived organisational support and employee diligence, commitment and innovation Journal of Applied Psychology 1990 75 51 59 10.1037/0021-9010.75.1.51

[B41] Eisenberger R Stinglhamber F Vandenberghe C Sucharski IL Rhoades L Perceived supervisor support: contributions to perceived organizational support and employee retention J Appl Psychol 2002 87 3 565 573 10.1037/0021-9010.87.3.565 12090614

[B42] Ohman-Strickland P Orzano AJ Nutting P Dickinson WP Scott-Cawiezell J Hahn K Gibel M Crabtree B Measuring organisational attributes of primary care practices: development of a new instrument Health Services Research 2007 42 3 1257 1273 10.1111/j.1475-6773.2006.00644.x 17489913 PMC1955254

[B43] Kotter J Leading change Why transformation efforts fail Harvard Business Review 2007

